# Myocardial infarction and haemorrhagic stroke as a rare presentation of acute aortic dissection: a fatal case report

**DOI:** 10.1093/ehjcr/ytad529

**Published:** 2023-10-24

**Authors:** Pablo Lasa-Berasain, Pablo Raposo Salas, Edurne Erice Azparren, Eva Regidor Sanz

**Affiliations:** Department of Critical Care Medicine, Navarre University Hospital, Irunlarrea 3, 31008, Pamplona, Navarre, Spain; Department of Cardiology, Navarre University Hospital, Irunlarrea 3, 31008, Pamplona, Navarre, Spain; Department of Critical Care Medicine, Navarre University Hospital, Irunlarrea 3, 31008, Pamplona, Navarre, Spain; Department of Critical Care Medicine, Navarre University Hospital, Irunlarrea 3, 31008, Pamplona, Navarre, Spain

**Keywords:** Acute aortic dissection, Acute myocardial infarction, Haemorrhagic stroke, Endovascular aortic repair, Case report

## Abstract

**Background:**

Type A acute aortic dissection (AAD) is an extremely severe condition, having a high risk of mortality. Initial diagnosis can be deceptive, especially in patients with other confounding presentations.

**Case summary:**

We present the case of a 60-year-old male with a history of endovascular aortic repair for abdominal aortic dissection, in whom a diagnosis of AAD was made, but almost missed, after he presented with stroke signs and left coronary myocardial infarction. Thorough clinical evaluation and point-of-care ultrasound (POCUS) were fundamental to the diagnosis of the underlying condition, which showed the intimal flap in the ascending aorta, aortic insufficiency, and a dissected left common carotid artery. The diagnosis was confirmed with a head and thoracic computed tomography scan, which also showed bilateral haemorrhagic strokes. Treatment options can be limited in patients with AAD with associated complications. After a careful multidisciplinary evaluation, life-sustaining therapy was withdrawn and the patient passed away.

**Discussion:**

Our case depicts the diagnosis challenge presented by patients with AAD. We emphasize the importance of clinical suspicion and POCUS examination for the diagnosis of the underlying condition, as it is frequently missed during first evaluation. We discuss the available literature regarding the prevalence and described mechanisms by which AAD can associate occlusion myocardial infarction, which more commonly involves the right coronary artery, as well as haemorrhagic stroke. We briefly mention management options, which are limited and controversial.

**Learning points**

- Acute aortic dissection (AAD) can present with a myriad of symptoms when complicated with malperfusion syndrome. Point-of-care ultrasound can help in the diagnosis when intimal flap image in the ascending aorta is present. The false lumen can also be identified in more distal arterial branches.
- Acute aortic dissection presenting with occlusion myocardial infarction is a rare complication that more commonly involves the right coronary artery.
- Ischaemic strokes are common under carotid involvement after AAD, but intraparenchymal haemorrhages are very infrequent and present a management challenge.

## Introduction

Acute aortic dissection (AAD) is a rare but lethal cardiac disease involving the aorta, which classically presents with sudden, severe chest pain radiating to the back.^1^ However, diagnosis can be challenging as initial presentation can frequently be atypical or mimic other pathologies when malperfusion syndrome is present.^2–6^ This arises when the false lumen of the AAD restricts or occludes flow in more distal branches of the aorta. It is not uncommon for the underlying diagnosis of AAD to be missed in the initial evaluation of these patients, which can impact management and prognosis.^7–10^ In this study, we report the case of a patient with a medical history of endovascular abdominal aortic aneurysm repair (EVAR), who presented with stroke signs and myocardial infarction that were both finally attributed to thoracic AAD (TAAD).

## Summary figure

**Figure:**
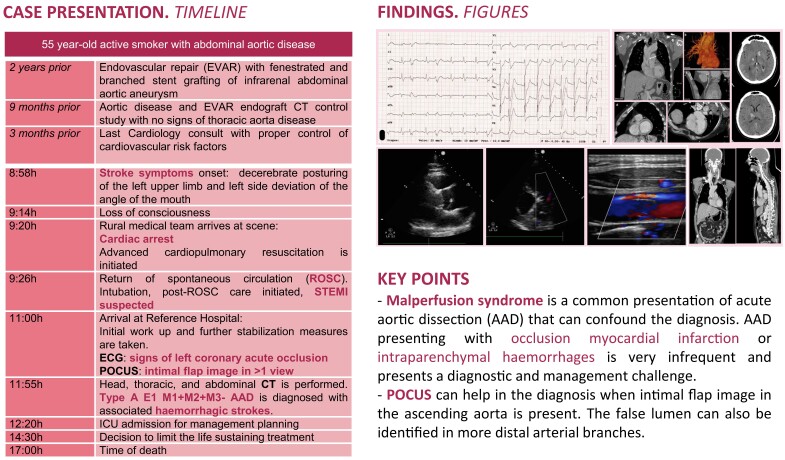


## Case summary

**Figure 1:**
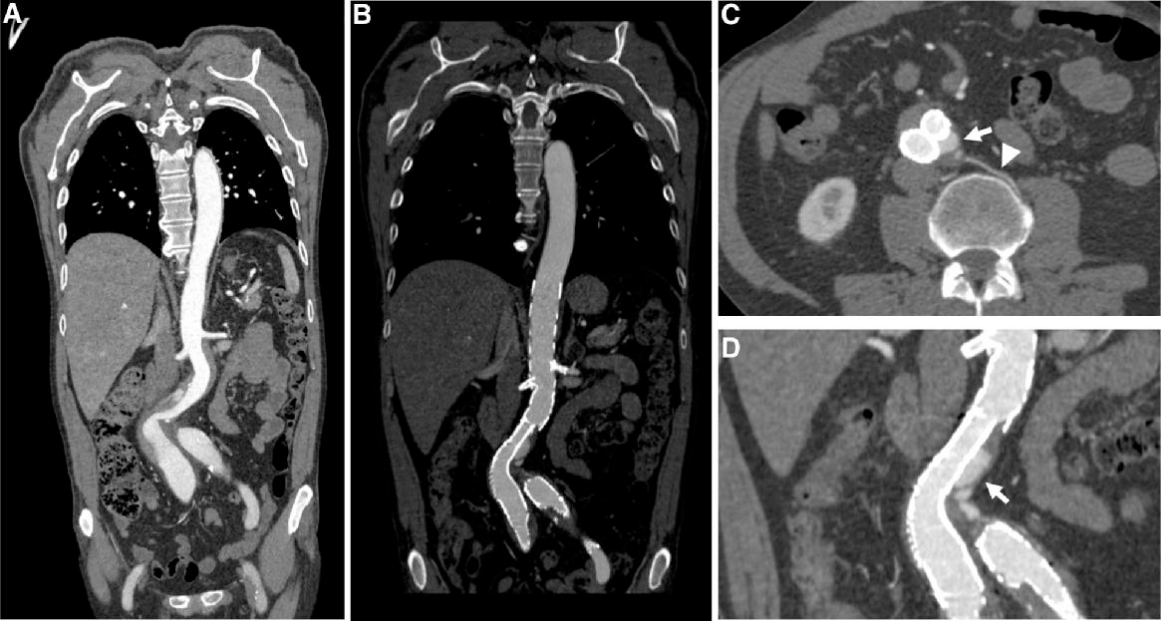
Computed tomography angiography studies of abdominal aorta disease. (*A*) Infrarenal abdominal aorta dissection extending to the right common iliac artery and bilateral common iliac artery aneurysms. (*B*) Endovascular abdominal aortic repair with branched and bifurcated custom-made endograft with Zone 4 Ishimaru’s proximal landing. (*C* and *D*) Type II distal graft endoleak (white arrows) due to backfilling of the aneurysm sac through a lumbar vessel (head arrow).

A 60-year-old Caucasian man unexpectedly presented a sudden onset of focal neurological deficit consisting of decerebrate posturing of the left upper limb and left side deviation of the angle of the mouth, followed by loss of consciousness. His medical history was significant for a 25 pack-year cigarette smoking history and EVAR procedure 2 years prior (*Figure 1A*), with follow-up computed tomography (CT) scan and transthoracic echocardiography studies done a year earlier showing no valvular heart disease, a 40 mm ascending aorta diameter with normal range diameter at the sinotubular junction and the sinuses of Valsalva, and a Type II distal EVAR graft endoleak (*Figure 1C* and *D*). The patient had an adequate control of cardiovascular risk factors through aspirin and statin therapy and no prior family history of aortopathy or known genetic risk factors. Upon the arrival of the first medical team, the patient suffered a cardiac arrest, following which advanced cardiopulmonary resuscitation was initiated, with the initial electrocardiogram (ECG) monitoring identifying a pulseless ventricular tachycardia. After two defibrillation shocks, eventually, spontaneous circulation was restored. The patient’s condition was marked by persistent haemodynamic instability, following which a norepinephrine drip was initiated. He was then transferred to the Reference Hospital by air ambulance under neurological sedation and invasive mechanical ventilation (IMV). However, information regarding his neurological symptoms in the initial presentation was lost in the process of data transmission to the air ambulance team.

**Figure 2:**
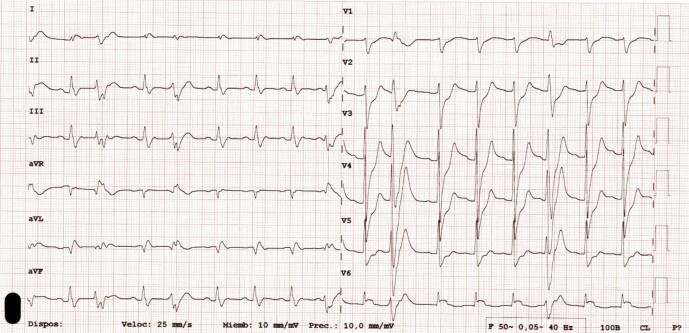
Twelve-lead electrocardiogram showing a sinus tachycardia, with a non-specific intraventricular conduction delay, and signs suggesting left anterior descending coronary artery acute occlusion with an ST depression with a prominent T wave in the anterior precordial leads (de Winter pattern) as well as a lateral ST elevation, most prominent in V6.

**Figure 3:**
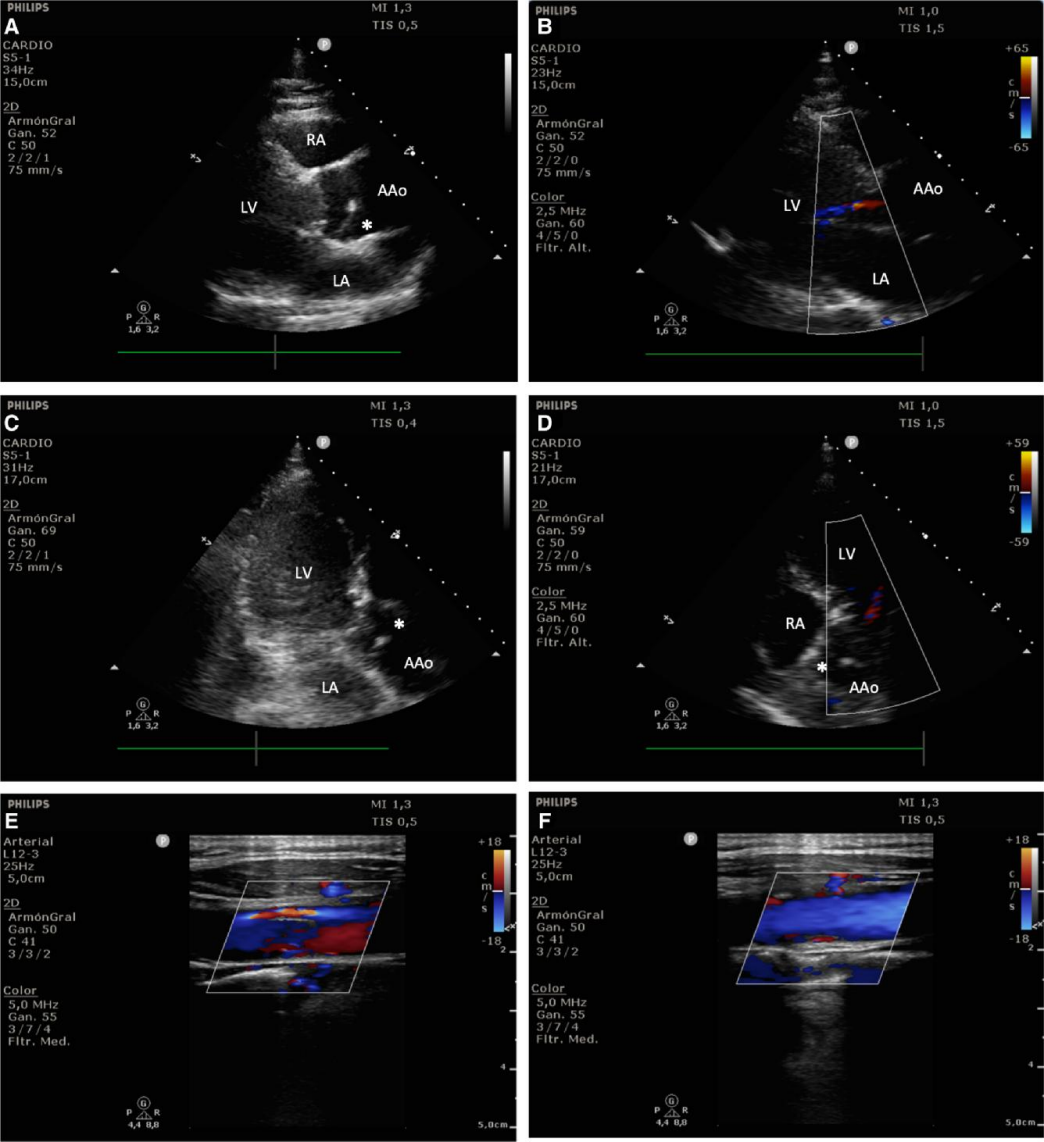
Point-of-care ultrasound images taken at admission. (*A*) Cardiac ultrasound parasternal long-axis plane showing the image of an intimal flap in the ascending aorta (*). (*B*) Doppler parasternal long axis showing a mild-to-moderate aortic insufficiency. (*C*) Apical three-chamber view showing the intimal image flap (*). (*D*) Apical five-chamber view where the intimal flap and aortic insufficiency are present. (*E*) Left common carotid artery with a false lumen and turbulent blood flow through the smaller true lumen. (*F*) Normal right common carotid artery. AAo, ascending aorta; LA, left atrium; LV, left ventricle; RA, right atrium.

**Figure 4:**
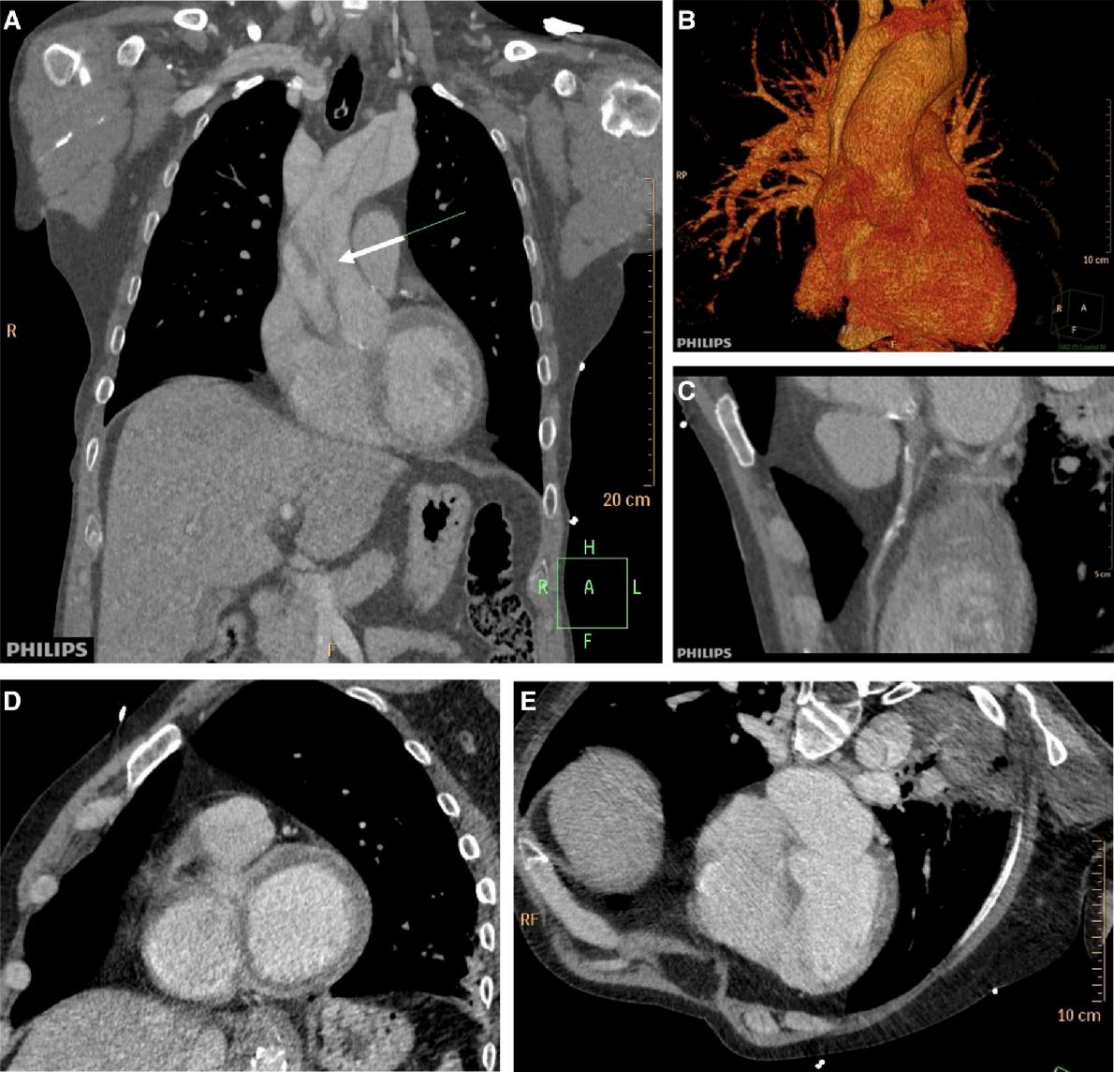
Chest computed tomography angiography showing acute Stanford Type A aortic dissection. (*A*) Coronal projection shows the entry tear in the ascending aorta (white arrow). (*B*) Volume rendering and (*C*) left coronary artery with calcified plaques. (*D* and *E*) Oblique sagittal and axial projections showing myocardial perfusion defects. Because of the poor quality of the study, the mechanism by which the left-main coronary ostium was involved in this case could not be described.

**Figure 5:**
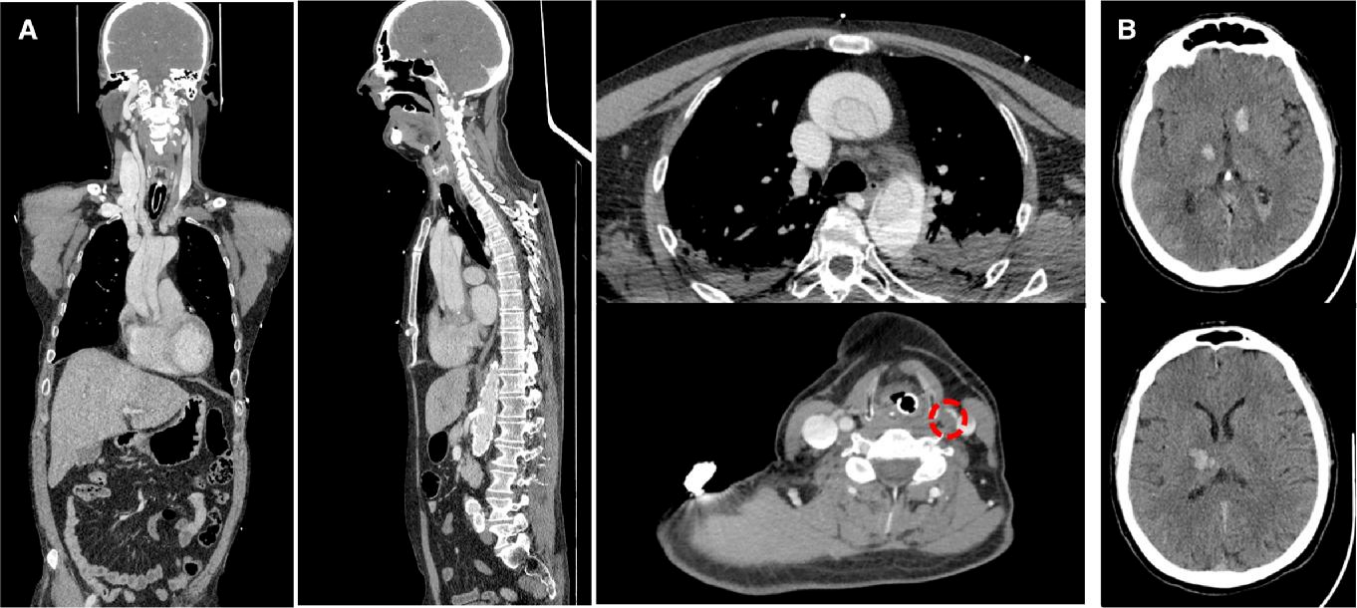
(*A*) Chest and neck computed tomography angiography showing a Stanford Type A acute aortic dissection extending from the ascending aorta to the descending aorta. The false lumen can be seen extending to the left common carotid artery as well (dotted circle). (*B*) Brain computed tomography scan showing two intraparenchymal haemorrhages at the basal ganglia bilaterally and another in between the right internal capsule and the thalamus.

On the patient’s admission to the Emergency Department, initial workup was performed, with physical examination showing signs of poor peripheral perfusion in the form of pale and cool extremities with augmented capillary refill time and weak but symmetrical distal pulses. At auscultation, early diastolic murmur was present in the aortic area, suggesting aortic regurgitation. Pupils were isochoric and normoreactive, and the remaining phases of neurological examination were restricted because of the influence of sedation medication. Arterial pressure was 86/67 mmHg with mild sinus tachycardia and a SaO_2_ 96% with a FiO_2_ of 100% on IMV. Electrocardiogram was performed, which showed an ST elevation in the lateral leads, which was most prominent in V6, and an ST depression with peaked T waves in the anterior precordial leads (the de Winter pattern), suggesting coronary occlusion myocardial infarction (OMI; *Figure 2*). At this point, the cardiac catheterization laboratory was notified under the suspicion of OMI involving the left anterior descending coronary artery. The patient was on long-term antiplatelet therapy, and no anticoagulation therapy was administered at this point, awaiting definite diagnosis. Arterial gas work showed lactic acidosis: pH 7.13 (normal range: 7.35–7.45), pCO_2_ 51 mmHg (normal range: 35–45), HCO_3_ 16.7 mmol/L (normal range: 22–26), lactate 5.45 mmol/L (normal range: 0.5–2.2). Blood work was not readably available at that moment but later revealed a normal Troponin I 3.3 pg/mL (normal value <4) and B-type natriuretic peptide 20 pg/mL (normal value <125), with a high D-dimer 31 800 ng/mL (normal value <500). Point-of-care ultrasound (POCUS) was suboptimal due to a poor acoustic window but sufficient to define a severe left ventricular dysfunction with a diffuse contractility alteration, which was more prominent in the lateral wall, which was consistent with the ECG findings. A more detailed inspection showed signs of possible intimal flap in the ascending aorta in multiple plane views (*Figure 3A* and *C*; Supplementary material online, *Video S1*), as well as aortic insufficiency, which was compatible with TAAD (*Figure 3B* and *D*; Supplementary material online, *Video S2*). Left common carotid artery (CCA) ultrasound showed signs of dissection with a prominent false lumen supporting the TAAD diagnosis (*Figure 3E*; Supplementary material online, *Video S3*), not present in the right CCA (see Supplementary material online, *Video S3*). After the patient’s condition stabilized, a chest CT angiography was performed, which showed a Stanford Type A AAD extending to the supra-aortic trunks (*Figures 4* and *5A*) and Type A E1 M1 + M2 + M3− dissection (TEM classification^7,8^). A brain CT scan was also performed, which showed intraparenchymal haemorrhages at the basal ganglia bilaterally and another in between the right internal capsule and the thalamus, suggestive of a hypertensive origin (*Figure 5B*). The GERAADA score predicted a 90.4% 30-day mortality risk after surgery. A multidisciplinary team that included a critical care specialist, an interventional cardiologist, a primary cardiologist, and a cardiac surgeon decided to limit the life-sustaining treatment, following which the patient expired. There was no prior family history, and immediate family members, who included eight siblings, showed no signs of aortic disease. Therefore, genetic testing was not performed.

## Discussion

Acute aortic dissection is an extremely severe condition with an incidence of 5–30 cases per million inhabitants per year, having a high risk of mortality ranging from 17 to 26%.^2,9,10^ Malperfusion syndrome with signs of myocardial ischaemia or neurological symptoms (among others) is not uncommon in these cases,^1,5,11^ but associated acute myocardial infarction (AMI) and haemorrhagic stroke, as depicted in this case report, are very rare.

### Acute aortic dissection and acute myocardial infarction

Around 10–20% of patients with AAD show signs of myocardial injury,^1,11–13^ but only 0.5–1% present with associated AMI.^14^ The involvement of the right coronary artery is far more common than the left branch.^3,14–17^ The proposed mechanisms by which AAD can cause coronary malperfusion are described as Type A, ostial coronary occlusion from the dissection flap, which more commonly originates from the right anterior aspect of the ascending aorta above the right sinus of Valsalva causing a trapdoor mechanism; Type B, an extension of the dissection into the coronary artery; and Type C, a complete circumferential detachment of the coronary artery with the dissection encircling it.^2,3^ In our patient, left coronary occlusion causing acute anterior MI was suspected, with the ECG and POCUS findings masquerading the underlying aetiology of AAD. In many cases, AAD diagnosis as the underlying condition can be missed and it can be performed during coronary angiography. Treatment is by surgery, but approaches with initial percutaneous coronary intervention with the aim of stabilizing the patient and reducing myocardial damage can be successful.^1,2,18^ Unfortunately, the mortality rate is high, and more studies are needed to define the most appropriate care for these patients.

### Acute aortic dissection and haemorrhagic stroke

Neurological manifestations are not infrequent in the initial presentation of AAD,^1^ appearing in up to 30% of patients,^5,6^ and can also hinder the diagnosis. Nevertheless, these symptoms are normally caused by perfusion impairment in the context of dissection extension to the supra-aortic trunks. In this scenario, AAD is responsible for ∼2% of ischaemic stroke presentations.^6^ In contrast, intraparenchymal haemorrhages are very rare, presenting in <0.5% of patients with AAD.^4,6^ Hypertension and atherosclerosis are risk factors for both diseases. However, the cause–effect relationship has not been well established.^4^ It is possible that a spike in systemic blood pressure at the time of an intracerebral haemorrhage may precipitate an AAD. This seemed more likely in our patient given the initial neurological onset and the bilateral presentation of the haemorrhages. Alternatively, the sympathetic stimulation and subsequent hypertensive crisis after an AAD can precipitate an intracerebral haemorrhage. This presentation can be deceptive, making the diagnosis and subsequent management a clinical challenge. Surgery for AAD complicated by brain haemorrhage is usually impossible because extracorporeal pump circulation equipment and anticoagulants cannot be used. In a few cases where a risk of immediate rupture of a AAD was deemed to be low, delaying the surgery and allowing the maturation of the intracranial haemorrhage have been successful.^4,6^ This approach was not possible in our patient due to the associated myocardial infarction that compromised haemodynamic stability.

### Thoracic acute aortic dissection after endovascular aortic repair

Iatrogenic TAAD is most common after cardiac surgery (43%) and endovascular cardiac interventions (40%). Aortic endovascular interventions account for only 7% of all reported cases of TAAD, and most data on EVAR-related TAAD were derived from TEVAR. Moreover, the majority of reported aortic dissections following EVAR is Type B. Type A AAD after EVAR, as reported in this case, is extremely rare, and only a few cases have been described in the literature.^19,20^ Moreover, TAAD manifested 2 years after the EVAR intervention, making it unlikely to be directly related to the procedure. Instead, it suggests a potential progression or extension of the aortic disease with an uncertain underlying aetiology.

### Ultrasound in acute aortic dissection

Careful evaluation is necessary in these scenarios to diagnose the underlying condition, and as the availability of POCUS examination increases, it is becoming an essential test. Point-of-care ultrasound in the form of focused cardiac ultrasonography (FCU) is a quick and simple test with a sensitivity rate of ∼75–85% in the visualization of the intimal flap, although the low negative predictive value does not rule out AAD.^21–23^ The aortic root and proximal ascending aorta are best imaged from the left parasternal long-axis view, and it may also be visualized in the apical three-chamber, apical five-chamber, and subcostal views. The typical feature is the image of a flap oscillation or motion that is independent of the aortic wall and visualized in more than one view (*Figure 3*), to distinguish it from artefacts secondary to reverberations,^21^ as well as an acute aortic regurgitation.^18^ Where FCU is suboptimal, it can also be helpful to extend the POCUS examination to more distal US-accessible arterial branches (*Figure 3E* and *F*; Supplementary material online, *Video S3*), where the false and true lumen can be recognized. Point-of-care ultrasound training and certification must be accredited to ensure access to proper education and maintain excellence in practice, as more medical professionals incorporate it to their clinical assessments conventionally based on physical examination alone.^24^

## Conclusion

We describe a catastrophic presentation of an AAD, presenting with associated anterolateral AMI and haemorrhagic stroke. These are two rare but known complications that can confound the diagnosis and severely impact treatment options. A careful evaluation of clinical presentation and diagnostic tools such as POCUS and FCU can help in the initial work-up evaluation. More studies are needed to define appropriate care for these patients.

## Supplementary Material

ytad529_Supplementary_Data

## Data Availability

The data underlying this article are available in the article and in its online Supplementary material.

## Lead author biography

Pablo Lasa-Berasain graduated from the University of Navarre in 2019 and is currently a resident in critical care medicine in the University Hospital of Navarre, Spain.

## Supplementary material

**Consent:** The patient’s next of kin has given full consent to the publication of this manuscript in accordance with COPE guidelines.

**Funding:** None declared.
